# Exploring functional and structural features of chemically related natural prenylated hydroquinone and benzoic acid from *Piper crassinervium* (Piperaceae) on bacterial peroxiredoxin inhibition

**DOI:** 10.1371/journal.pone.0281322

**Published:** 2023-02-24

**Authors:** Vitoria Isabela Montanhero Cabrera, Gabrielle do Nascimento Sividanes, Natalia Fernanda Quintiliano, Marcos Hikari Toyama, João Henrique Ghilardi Lago, Marcos Antonio de Oliveira

**Affiliations:** 1 Instituto de Biociências, Universidade Estadual Paulista, UNESP, São Vicente, SP, Brazil; 2 Centro de Ciências Naturais e Humanas, Universidade Federal do ABC, Santo André, SP, Brazil; University of East Anglia, UNITED KINGDOM

## Abstract

Multiple drug resistance (MDR) bacterial strains are responsible by 1.2 million of human deaths all over the world. The pathogens possess efficient enzymes which are able to mitigate the toxicity of reactive oxygen species (ROS) produced by some antibiotics and the host immune cells. Among them, the bacterial peroxiredoxin alkyl hydroperoxide reductase C (AhpC) is able to decompose efficiently several kinds of hydroperoxides. To decompose their substrates AhpC use a reactive cysteine residue (peroxidatic cysteine—Cys_P_) that together with two other polar residues (Thr/Ser and Arg) comprise the catalytic triad of these enzymes and are involved in the substrate targeting/stabilization to allow a bimolecular nucleophilic substitution (S_N_2) reaction. Additionally to the high efficiency the AhpC is very abundant in the cells and present virulent properties in some bacterial species. Despite the importance of AhpC in bacteria, few studies aimed at using natural compounds as inhibitors of this class of enzymes. Some natural products were identified as human isoforms, presenting as common characteristics a bulk hydrophobic moiety and an α, β-unsaturated carbonylic system able to perform a thiol-Michael reaction. In this work, we evaluated two chemically related natural products: 1,4-dihydroxy-2-(3’,7’-dimethyl-1’-oxo-2’*E*,6’-octadienyl) benzene (**C1**) and 4-hydroxy-2-(3’,7’-dimethyl-1’-oxo-2’*E*,6’-octadienyl) benzoic acid (**C2**), both were isolated from branches *Piper crassinervium* (Piperaceae), over the peroxidase activity of AhpC from *Pseudomonas aeruginosa* (PaAhpC) and *Staphylococcus epidermidis* (SeAhpC). By biochemical assays we show that although both compounds can perform the Michael addition reaction, only compound **C2** was able to inhibit the PaAhpC peroxidase activity but not SeAhpC, presenting IC_50_ = 20.3 μM. SDS-PAGE analysis revealed that the compound was not able to perform a thiol-Michael addition, suggesting another inhibition behavior. Using computer-assisted simulations, we also show that an acidic group present in the structure of compound **C2** may be involved in the stabilization by polar interactions with the Thr and Arg residues from the catalytic triad and several apolar interactions with hydrophobic residues. Finally, **C2** was not able to interfere in the peroxidase activity of the isoform Prx2 from humans or even the thiol proteins of the Trx reducing system from *Escherichia coli* (EcTrx and EcTrxR), indicating specificity for *P*. *aeruginosa* AhpC.

## Introduction

Bacteria resistance to antibacterial drugs may be directly related to oxidative stress since antibiotics from different classes have the common ability to generate reactive oxygen species (ROS), damaging macromolecules, especially DNA [1, 2]. ROS are also produced by host immune cells to fight pathogen infections, and studies suggest that inhibition of antioxidant enzymes is deleterious to pathogens infection and establishment [3]. Bacteria cells possess several antioxidant enzymes able to decompose ROS, including the typical 2-Cys peroxiredoxins, so called AhpCs [4]. These enzymes are very abundant in bacteria and are considered virulence factors to some species, indicating they play an important role in pathogenesis [3, 5–10]. AhpC is the abbreviation used for alkyl hydroperoxide reductase subunit C, referring to its discovery, since the bacterial strains carrying deletions of *ahpC* gene were very sensitive to organic hydroperoxides [11]. Later studies revealed that AhpC are also very reactive to several kinds of hydroperoxide, such as hydrogen peroxide (H_2_O_2_) and peroxynitrite (NOO^-^) [12–14].

**Fig 1:**
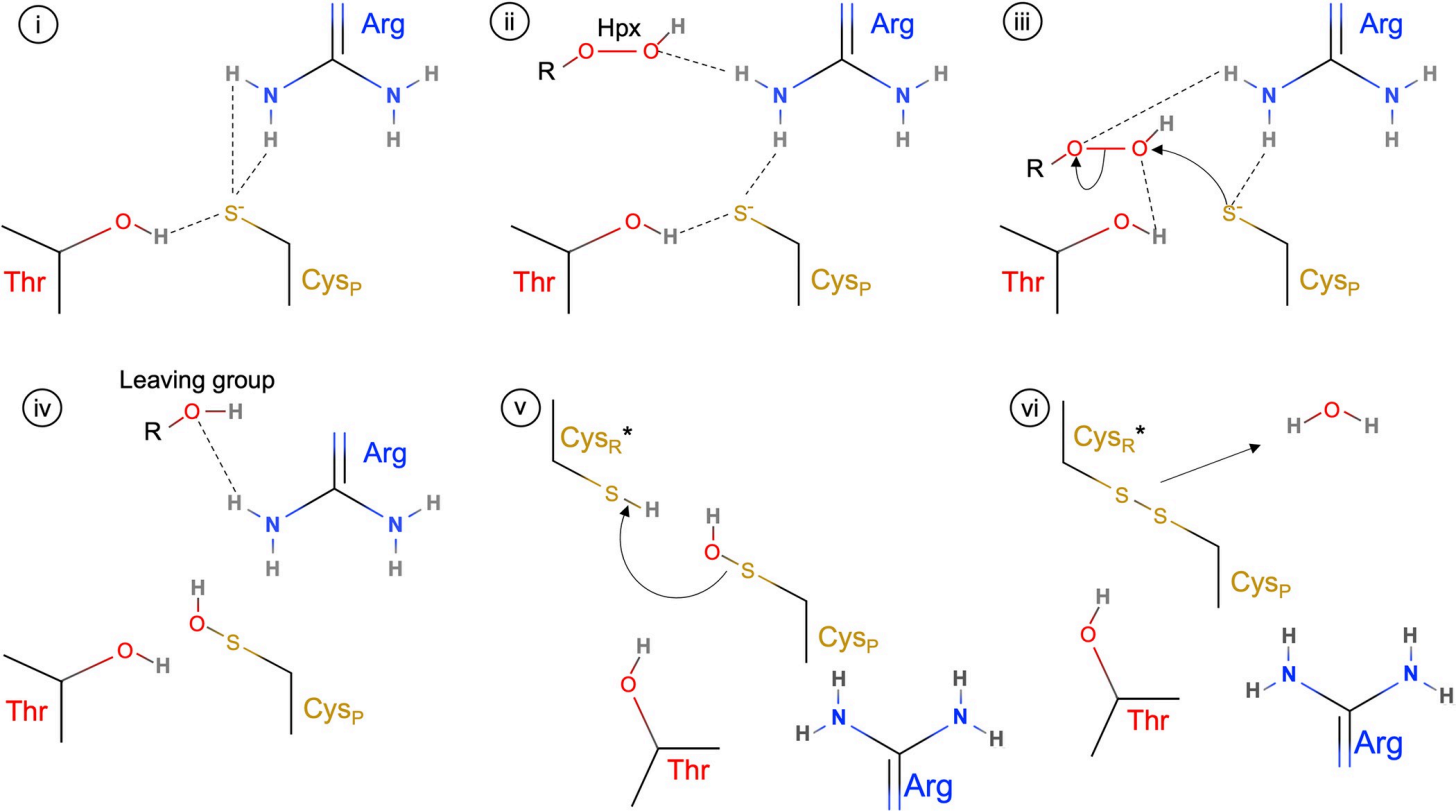
Molecular interactions and peroxide decomposition reactions of 2-Cys Prx. The thiolate nucleophilicity of peroxidatic cysteine (Cys_P_) is increased by hydrogen bonds with the catalytic triad Arg and Thr residues **(i)**. The hydroperoxide (Hpx) is trapped by the Arg hydrogen bond which is able to target the Hpx to the Prx active site **(ii)**. The shift of the Arg and Thr hydrogen bonds from the Cys_P_ thiolate to the substrate stabilizes the Hpx and increase the thiolate reactivity enabling the S_N_2 mechanism **(iii)**. After the catalytic reaction, the Cys_P_ is oxidized to cysteine sulfenic acid (Cys_P_-SOH) and the release of the leaving group (R-OH, meaning a water molecule in the case of hydrogen peroxide, or the alcohol derivative, in the case of organic hydroperoxides) is assisted by polar interaction with Arg **(iv**). The cysteine sulfenic acid formation triggers structural changes which allow the condensation among Cys_P_-SOH with Cys_R_-SH **(v)** resulting in the disulfide formation and concomitant release of a water molecule **(vi)**. The asterisk (*) in Cys_R_, denotes the adjacent subunit of the homodimer.

The peroxidatic mechanism of typical 2-Cys Prx is based on a conserved catalytic triad (TC) composed of a reactive cysteine residue (peroxidatic cysteine—Cys_P_), an Arg, and a Thr or Ser. The mechanistic importance of the catalytic triad residues has been well studied experimentally, and it is based on an intricate hydrogen bonds network involved in the hydroperoxide decomposition [15, 16]. The Arg and Thr/Ser are able to stabilize the Cys_P_ in the thiolate state (Cys_P_-S^-^) by hydrogen bonds, decreasing its p*K*_a_. These residues are also involved in the orientation and activation of the hydroperoxide molecule (R-OOH) by hydrogen bonds network, allowing the optimal reactivity of Cys_P_ through a S_N_2 mechanism [15, 16]. Together, this molecular mechanism culminates in Cys_P_ oxidation to cysteine sulfenic acid (CysP—SOH) and the release of the leaving group (R-OH) (**Fig 1**). Computer-assisted simulations of the reaction of different Prxs with H_2_O_2_ gave further insights into the mechanism of catalysis at a molecular level, supporting the catalytic mechanism proposed [17, 18].

The typical 2-Cys Prx are obligate homodimers. After hydroperoxide decomposition, the Cys_P_ forms an intramolecular disulfide with a second cysteine residue (resolving Cys; Cys_R_) found in the adjacent subunit. Despite that, the minimum catalytic unit of 2-Cys Prx has been homodimer, when these enzymes are in the reduced state (Cys_P_-S^-^) they are found as α 2 [5] decamers, forming a structure that resembles a doughnut [20]. In enzymes containing Thr as part of the catalytic triad, the peroxide decomposition with consequent oxidation of the cysteines to disulfide triggers conformational changes that result in the decamer disruption in dimers [19–21]. Conversely to 2-Cys Prx containing Ser in the CT, the disulfide formation occurs without significant quaternary structure transition [22, 23]. The reducing agent of typical 2-Cys Prx are represented by the thioredoxin system or by the AhpF which are able to reduce the Prx disulfide, restoring the reactive state [23, 24].

Despite the apparent AhpC importance to the establishment and survival of pathogenic bacteria in hosts, few studies aimed at finding inhibitors to these enzymes [25, 26]. Some isoforms of typical Prx 2-Cys are overexpressed in some human genetic diseases, including cancer and biological processes as inflammation, and some inhibitory macro and micro-molecules, such as antibodies and natural or synthetic low molecular weight compounds, have already been characterized as eukaryotic counterparts [27–34]. A common feature shared among low molecular weight compounds is a very bulky carbon skeleton, which may resemble natural oxidant substrates as organic hydroperoxides. Among the natural compounds identified to eukaryotic isoforms, three of them are *ent*-kauranes diterpenes (adenanthin, JM-202, and parvifoline AA). The inhibitory action of these compounds is based on the alkylation of the catalytic cysteines through a thiol-Michael addition involving an α,β-unsaturated carbonylic system from the inhibitors [29, 30, 33].

As part of our continuous studies concerning the identification of natural inhibitors of peroxiredoxins, we here isolated two chemically related natural compounds– 1,4-dihydroxy-2-(3’,7’-dimethyl-1’-oxo-2’*E*,6’-octadienyl) benzene (**C1**) and 4-hydroxy-2-(3’,7’-dimethyl-1’-oxo-2’*E*,6’-octadienyl) benzoic acid (**C2**) from branches of *Piper crassinervium* (Piperaceae). These chemicals were tested over the AhpCs containing Thr or Ser as part of the catalytic triad, using recombinant enzymes from *Pseudomonas aeruginosa* and *Staphylococcus epidermidis*, opportunistics Gram -negative and -positive bacteria involved in hospital acquired infections. Using biochemical approaches, we identified compound **C2** as a novel natural product with inhibitory properties over PaAhpC but not over SeAhpC, that presents IC_50_ in the order of 20.3 μM. We also demonstrated that the inhibitory properties were not due to the thiol-Michael addition mechanism over the catalytic cysteines, since the intramolecular disulfide formation of the enzyme was preserved. We also evaluated the inhibitory effect of the compound **C2** over the human isoform Prx2 and the thiol proteins Trx and TrxR from bacteria, and no significant inhibition was detected in the tested conditions, suggesting specificity to PaAhpC. Finally, we executed computer assisted molecular simulations to investigate a possible mode of binding, which revealed that compound **C2** is maintained in the active site by several non-polar interactions with residues of the PaAhpC active site microenvironment comprising three subunits of the decamer. Curiously **C2** possess physicochemical characteristics that resemble organic peroxides found in biological systems that are derived from aromatic compounds or long chain molecules, such as nitrogenous bases and lipids hydroperoxides and, ultimately, may mimics these kinds of biological substrates. Altogether, our results reveal a novel class of inhibitory natural compounds able to exert activity over the bacterial PaAhpC.

## Experimental

### General

Chromatographic separation procedures were performed using silica gel (Merck, 230–400 mesh) and silica gel 60 PF_254_ (Merck) for column separation and for analytical (0.50mm) TLC, respectively. NMR spectra were recorded on a Varian Inova 500 spectrometer, operating at 500 MHz (^1^H nuclei) and 125 MHz (^13^C nuclei). CDCl_3_ (Aldrich) was used as a solvent whereas tetramethylsilane (TMS) was employed as an internal standard. ESI-HRMS were recorded on a Bruker Daltonics MicroTOF QII spectrometer acquired with ESI (electrospray ionization) in both positive and negative ion mode. All solvents and reagents were purchased from Sigma-Aldrich (St. Louis, MO, USA) and Thermo Scientific (Logan, UT, USA).

### Plant material

Branches of *P*. *crassinervium* were collected at Parque Estadual Fontes do Ipiranga, São Paulo State, Brazil and identified by Dr. Guilherme M. Antar from the Instituto de Biociências of Universidade de São Paulo (IB-USP), receiving the registration code SISGEN A4123E4. A voucher specimen (SPF 218827) was deposited in the Herbarium of IB-USP, São Paulo, SP, Brazil.

### Extraction and isolation of the constituents

Dried and powdered branches (85 g) were exhaustively extracted with *n*-hexane at room temperature. The solvent of combined *n*-hexane was evaporated under reduced pressure to afford 747 mg of crude extract. Part of this material (670 mg) was chromatographed over a silica-gel column, eluted with increasing amounts of EtOAc in *n*-hexane, to afford pure **C1** (79.5 mg) and **C2** (44.2 mg).

### Recombinant proteins

*P*. *aeruginosa* AhpC (PaAhpC), *S*. *epidermidis* AhpC (SeAhpC), and the Trx system from *Escherichia coli* (EcTrx and EcTrxR) were previously obtained [22]. The expression of the human Prx2 (HsPrx2) and *Saccharomyces cerevisiae* Trx system (ScTrx1 and ScTrxR1) were performed as previously described by Truzzi and colleagues [35].

### Protein expression and purification

Transformed *E*. *coli* BL21 (DE3) cells containing pET15b-*pa_ahpc*, pET15b-*se_ahpc*, pET15b-*ec_trx*, pET15b-*ec_trxr*, pET28b-*hs_*prx2, pET15b-*sc_trx1* and pPROEX-*sctrxr1* were grown overnight at 37°C in 100 mL of medium LB (10% tryptone, 5% NaCl and 5% yeast extract) containing 100 μg/mL of ampicillin and then transferred to 1 L of fresh LB containing ampicillin (100 μg/mL) and grown to OD_600_ = 0.6–0.8. Recombinant protein expression was induced by IPTG addition to a final concentration of 1 mM for 3 hours at 37°C. The cells were harvested by centrifugation at 4.000 × *g*/4°C/20 min, and the pellet was suspended with start buffer (20 mM sodium phosphate pH 7.4; 300 mM NaCl; 20 mM imidazole) containing PMSF (1 mM). Cell disruptions were performed by sonication (30% amplitude/ 24 cycles of 5 seconds and resting for 15 seconds in an ice bath, and nucleic acids were removed using streptomycin sulfate ([Final] = 1%). The suspensions were clarified by filtration (45μm membrane; Merck–Millipore) and following centrifugation (45 min/4°C/12.000 g) and the protein extracts free of nucleic acids were collected.

### Determination of enzyme concentrations

Protein concentrations were measured by spectrophotometry at λ = 280 nm by the molar extinction coefficient using the ProtParam tool (http://web.expasy.org/protparam). The enzyme abbreviations, Uniprot codes and enzyme properties are presented in Table 1.

**Table 1:** Molar extinction coefficients, molecular weight and Uniprot code of the proteins used in this work.

Enzyme	ε 280 nm (M^−1^ cm^−1^)	Molecular weight (kDa)	Uniprot entry
PaAhpC	21095	20.54	Q9I6Z3
SeAhpC	25556	21.08	Q8CMQ2
EcTrx	14105	12.63	P0AA25
EcTrxR	19160	35.45	P0A9P4
HsPrx2	21555	21.89	P32119
ScTrx1	10095	11.23	P22217
ScTrxR1	27640	34.23	P29509

Abbreviations: PaAhpC = *P*. *aeruginosa* AhpC, SeAhpC = *S*. *epidermidis* AhpC, EcTrx = *E*. *coli* Trx1; EcTrxR = *E*. *coli* TrxR1, HsPrx2 = *H*. *sapiens* Prx2, ScTrx1 = *S*. *cerevisiae* Trx1 and ScTrxR1 = *S*. *cerevisiae* TrxR1.

### Treatment of the enzymes with compounds C1 and C2

Enzyme samples [100 μM] were reduced with 5mM dithiothreitol (DTT) for 30 minutes at 25°C and desalted using PD10 columns (Cytiva Life Sciences, Marlborough, MA, USA), quantified, and the protein concentration were adjusted to 10 μM and treated with 50 M equivalents of compounds **C1** or **C2** for one hour at room temperature. As control of cysteine alkylation/enzyme inactivation, the Prx were treated with N-ethylmaleimide (NEM) 1h/RT. Finally, the treated proteins were desalted again to remove excess of the compounds and quantified again for use in subsequent assays.

### NADPH coupled oxidation assay to test inhibitory properties of compounds C1 and C2

The inhibitory properties of compounds **C1** and **C2** on PaAhpC and SeAhpC peroxidase activity were tested using the *E*. *coli* Trx system by the coupled NADPH oxidation assay as previously described [22]. Briefly: 3 μM of PaAhpC or SeAhpC was added to a mix containing 6 μM of EcTrx, 0.9 μM of EcTrxR, 150 μM of NADPH, in 50 mM HEPES pH 7.4 buffer, 1 mM DTPA and 100 μM sodium azide, and then incubated for 5 minutes at 37°C. The reactions were initiated by the addition of H_2_O_2_ (500 μM) and NADPH decay were monitored spectrophotometrically at 340 nm. As negative controls are represented by reactions without AhpC are (negative control), or AhpC pretreated with NEM (40 μM) (inhibition control), proteins without pretreatment (positive control), and proteins pretreated either with compounds **C1** and **C2**. To evaluate the inhibitory activity of compound **C2** over the human peroxiredoxin isoform were used human HsPrx2 (5 μM) and the heterologous yeast Trx system (ScTrx1, 10 μM and ScTrxR1, 0.2 μM).

### Analysis of protein sequences and crystallographic structures

Protein alignments were performed using Clustal Ω [36], and the graphical representations were generated using JalView [37]. Molecular crystallographic structures representations were made using the PyMol (https://pymol.org/2/).

### Molecular docking approaches

Molecular dockings were performed using the coordinates 4MA9 from *S*. *typhimurium* AhpC obtained from the Protein Data Bank. Three-dimensional structures of compounds **C1** and **C2** were constructed using MolView [38]. Docking simulations were performed using AutoDock Vina [39, 40], griding the active site region (20 × 20 × 20Å), using the deprotonates Cys_P_ (thiolate state). Were generated 15 positions per simulation. Each ligand orientation was analyzed using UCSF Chimera tool [41] to determine the best pose using as criterion the distance of the reactive Cys_P_ and the α,β-unsaturated group of the compounds with maximum distance of 5.0 Å and also the position compared with ligands found in the crystallographic structures of Prx isoforms active site as H_2_O_2_ (PDB code = 3A2V), benzoate (2V32 1HD2; 1OC3; 2V41; 1H4O) and tert-butylbenzene-diol (4K7O). The protein-ligand analysis interactions of the best poses were performed using the LigPlot+.

## Results

### Chemical characterization of compounds C1 and C2

**Fig 2:**
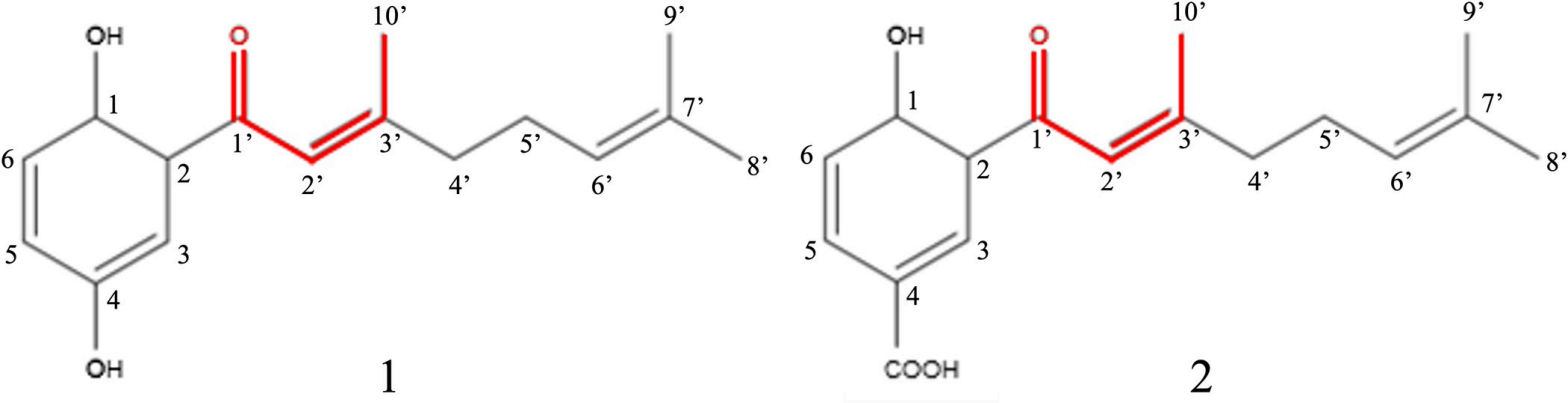
Chemical structures of compounds C1 and C2, isolated from branches of *P*. *crassinervium*. The α, β-unsaturated carbonyl system are represented in red.

Compounds **C1** and **C2**, isolated as white amorphous solids, displayed [M–H]^-^ ion peaks in ESI-HRMS at *m/z* 259.1328 and 287.1322, respectively. ^1^H NMR spectra indicated the presence of 1,2,4-trisubstituted aromatic ring based on the signals at δ 6.84 (d, *J* = 8.9 Hz, H-6), 6.98 (dd, *J* = 8.9 and 3.0 Hz, H-5) and 7.21 (d, *J* = 3.0 Hz, H-3) for compound **C1,** and at δ 7.03 (d, *J* = 8.7 Hz, H-6), 8.15 (dd, *J* = 8.7 and 1.6 Hz, H-5) and 8.58 (d, *J* = 1.6 Hz, H-3) for compound **C2**. These different signals suggest the occurrence of hydroquinone and 4-hydroxylbenzoic unities in compounds **C1** and **C2**, respectively, as previously reported in metabolites isolated from leaves of *P*. *crassinervium* [42]. Despite these differences, ^1^H NMR spectra of both compounds showed similar signals attributed to 1-oxo-geranyl moiety at δ 1.62 (H-9’), 1.72 (s, H-8’), 2.18 (s, H-10’), 2.30 (m, H-4’), 2.20 (m, H-5’), 5.12 (t, *J* = 7.3 Hz), and 6.66 (s, H-2’). ^13^C NMR spectra of compounds **C1** and **C2** exhibited signals attributed to sp^2^ carbons of geranyl moiety at δ 196.1/195.8 (C-1’), 115.0/118.8 (C-2’), 161.4/163.8 (C-3’) 124.2/122.7 (C-6’), and 132.9/133.0 (C-7’), as well as three methyl groups at δ 25.7 (C-8’), 17.8 (C-9’), and 20.1/20.4 (C-10’); and two methylene carbons at δ 41.6/41.9 (C-4’) and 26.2/26.3 (C-5’). In the case of ^13^C NMR spectrum of compound **C1,** the remaining six peaks were observed at δ 119.2 (C-1), 119.7 (C-2), 120.6 (C-3), 123.0 (C-4), 147.3 (C-5), and 157.4 (C-6), peaks that were attributed to carbons of a hydroquinone system. For compound **C2**, seven signals of sp^2^ carbons, assigned to 4-hydroxybenzoic unit, were observed at δ 119.8 (C-1), 167.7 (C-2), 118.9 (C-3), 137.0 (C-4), 120.2 (C-5), 133.1 (C-6), and 170.5 (COOH) (**S1–S6 Figs**). Comparison with literature data allowed the identification of compounds **C1** and **C2** as 1,4-dihydroxy-2-(3’,7’-dimethyl-1’-oxo-2’*E*,6’-octadienyl) benzene and 4-hydroxy-2-(3’,7’-dimethyl-1’-oxo-2’*E*,6’-octadienyl) benzoic acid [42, 43], respectively (**Fig 2**).

### Evaluation of inhibitory properties of compounds C1 and C2

**Fig 3:**
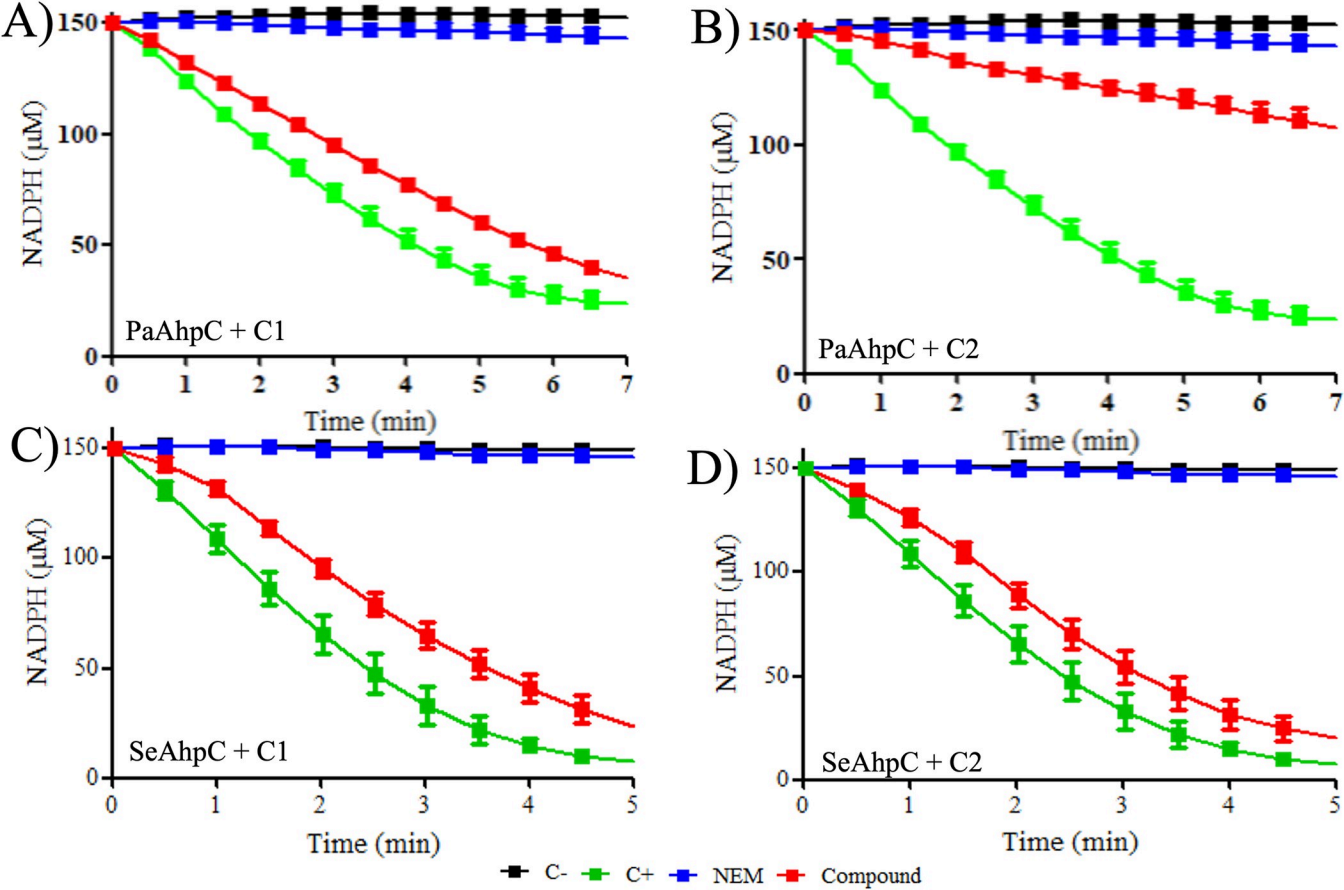
NADPH oxidation assay of PaAhpC and SeAhpC after treatment with compounds C1 and C2. To perform the assays PaAhpC and SeAhpC samples were previously reduced with DTT (5 mM/1h). The DTT excess was removed, and the enzymes were treated with 40 molar equivalents of **C1** (**A** and **C**, red square) or **C2** (**B** and **D**, red square) for 1hour at room temperature. The compounds excess was removed, and the peroxidase activity was accessed by the NADPH oxidation in reactions containing: 3 μM of PaAhpC or SeAhpC, 6 μM EcTrx, 0.9 μM EcTrxR and 150 μM NADPH in buffer 50 mM HEPES (pH = 7.4), 100 μM DTPA and 1 mM sodium azide. Reactions were incubated at 37°C for 5 minutes before being initiated by the addition of H_2_O_2_ (500 μM) and monitored spectrophotometrically at 340 nm, 37°C, for 5 minutes. Reactions containing PaAhpC or SeAhpC without natural compounds treatment (green square) were used as positive controls for peroxidase activity, and reactions without AhpC (black square) were used as negative controls. PaAhpC or SeAhpC samples previously treated with NEM were used as control of protein inhibition (blue square). All experiments were performed at least three times in triplicate.

To assess the inhibitory activity of compounds **C1** and C2, we employed the NADPH oxidation assay [29] using the recombinant AhpC from *P*. *aeruginosa* (PaAhpC) or *S*. *epdidermidis* (SeAhpC) and the heterologous Trx system from *E*. *coli*. Representative results of the PaAhpC, SeAhpC, EcTrx, and EcTrxR expression and purification procedures are shown in **S7A–S7D Fig**. As inhibition control, reduced samples of PaAhpC or SeAhpC were treated with NEM, a well-known and powerful cysteine alkylating agent that is able to annihilate peroxiredoxin activity [44]. As expected for the samples subjected to NEM treatment, no peroxidase activity was detected to both enzymes as indicated by the initial rates of hydrogen peroxide decomposition when compared to the enzyme without treatment (**Fig 3 and Table 2**).

**Table 2:** Initial rates of hydrogen peroxide decomposition by PaAhpC or SeAhpC after treatment with compounds C1 or C2.

Sample	No enzyme (Negative control)	Enzyme + NEM (Inhibition control)	Enzyme (Positive control)	Enzyme + C1			Enzyme + C2
PaAhpC *v*_0_ (μMs^-1^)	0.02 ± 0.01	0.04 ± 0.01	0.52 ± 0.02		0.46 ± 0.01	0.13 ± 0.03	
SeAhpC *v*_0_ (μMs^-1^)	0.01 ± 0.01	0.03 ± 0.01	0.70 ± 0.07		0.65 ± 0.02	0.60 ± 0.01	

Abbreviations: PaAhpC = *P*. *aeruginosa* AhpC and SeAhpC = *S*. *epidermidis* AhpC.

Regarding the tested natural products, compound **C1** was not able to interfere significatively on the peroxidase activity of both AhpCs (**Fig 3A and 3C; Table 2**). Conversely, while the compound **C2** nearly abolished PaAhpC enzyme activity, no significant effect was observed on SeAhpC (**Fig 3B and 3D; Table 2**). We also compared the residual amounts of NADPH after 300 seconds of reaction, and the results confirmed that compound **C2** was able to inhibit the peroxidase activity of PaAhpC but not of SeAhpC (**S8 Fig**).

The difference in the inhibitory properties of compound **C2** over the AhpCs of *P*. *aeruginosa* and *S*. *epidermidis* may rely on differences in enzyme structure. It has been shown that PaAhpC and SeAhpC present distinct properties including redox structural switches (dimer → decamer transition) and peroxidase activity over distinct hydroperoxides which were correlated to the single amino acid substitution in the catalytic triad [22], suggesting that structural peculiarities may impact the interaction with the substrates.

### Determination of IC_50_ of compound C2 to PaAhpC

**Fig 4:**
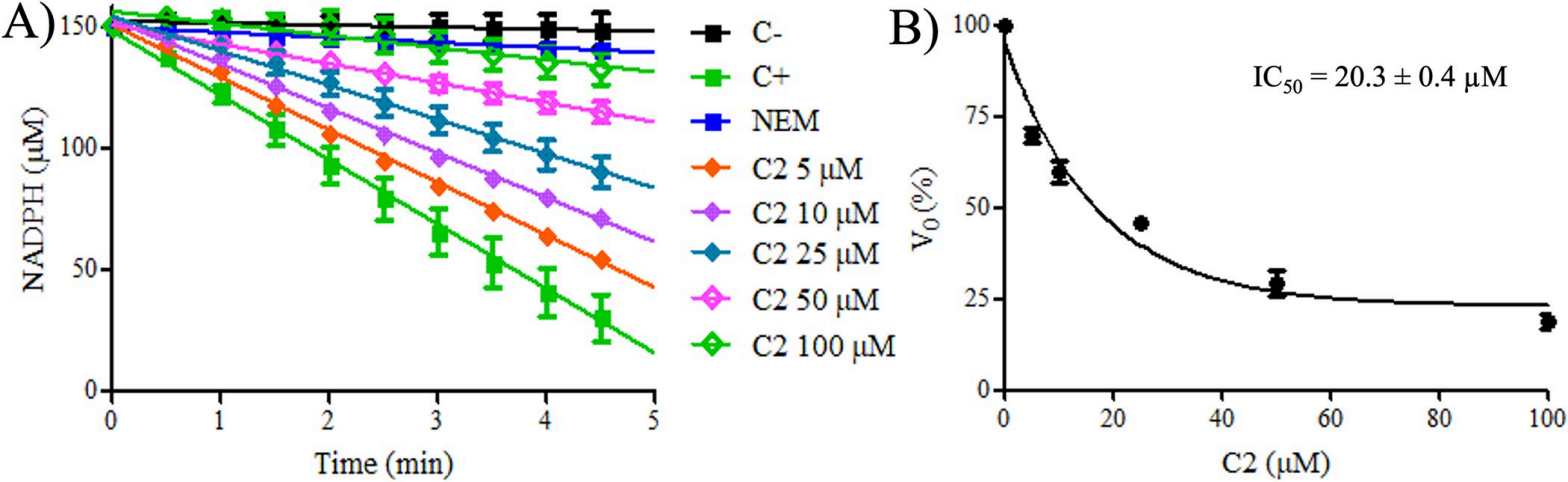
IC_50_ determination of compound C2 for PaAhpC. **(A)** Aliquots of PaAhpC enzyme were reduced with 5 mM DTT for 1 hour and then desalted to remove excess of reductant. Aliquots containing 10 μM of the reduced and desalted enzymes were treated varying the concentration of compound **C2** (5, 10, 25, 50 and 100 μM for 1 hour at room temperature and subsequently desalted again. Next, 3μM of the treated proteins were added to reactions containing 6 μM EcTrx, 0.9 μM EcTrxR, 150 μM NADPH, 50 mM HEPES (pH 7.4), 100 μM DTPA and 1 mM sodium azide. The reactions were incubated at 37°C for 5 minutes before being initiated by the addition of 500 μM H_2_O_2_ and monitored spectrophotometrically at 340 nm, 37°C, for 5 minutes. Reactions without treatment with compound **C2** were used as positive controls (green square), and reactions without the addition of AhpC were used as negative controls (black square). **(B)** The initial rates (*v*_0_) of each reaction were calculated, transformed into percentual values, and plotted to calculate the IC_50_ values of compound **C2** for PaAhpC. All experiments were performed at least three times in triplicate.

Since the compound **C2** was able to inhibit the peroxidase activity of PaAhpC, we proceeded to determine the apparent IC_50_ of the enzyme. For this, previously reduced enzymes samples were treated with different stoichiometric ratios of compound **C2** (5, 10, 25, 50, and 100 molar equivalents). Excess was removed and the PaAhpC was evaluated by the NADPH oxidation assay (**Fig 4A**). Our results revealed that the amount of compound **C2** necessary to inhibit the PaAhpC was approximately 20 μM (**Fig 4B**), a high value for an inhibitor but similar to the amount of the compound adenanthin (15 μM), another natural inhibitor to the human Prx2 [29].

### Evaluation of specificity over the Prx human isoform and oxidoreductases thiol proteins

Since compound **C2** did not exert a significant inhibitory effect on SeAhpC, we evaluated if the compound was able to inhibit other thiol proteins. Initially, we evaluated the inhibitory effect over Prx2 from humans (HsPrx2), the host of *P*. *aeruginosa*, and Trx and TrxR from bacteria, since some inhibitors described to eukaryotic Prx also inhibit the two latter thiol proteins [26, 45, 46].

**Fig 5:**
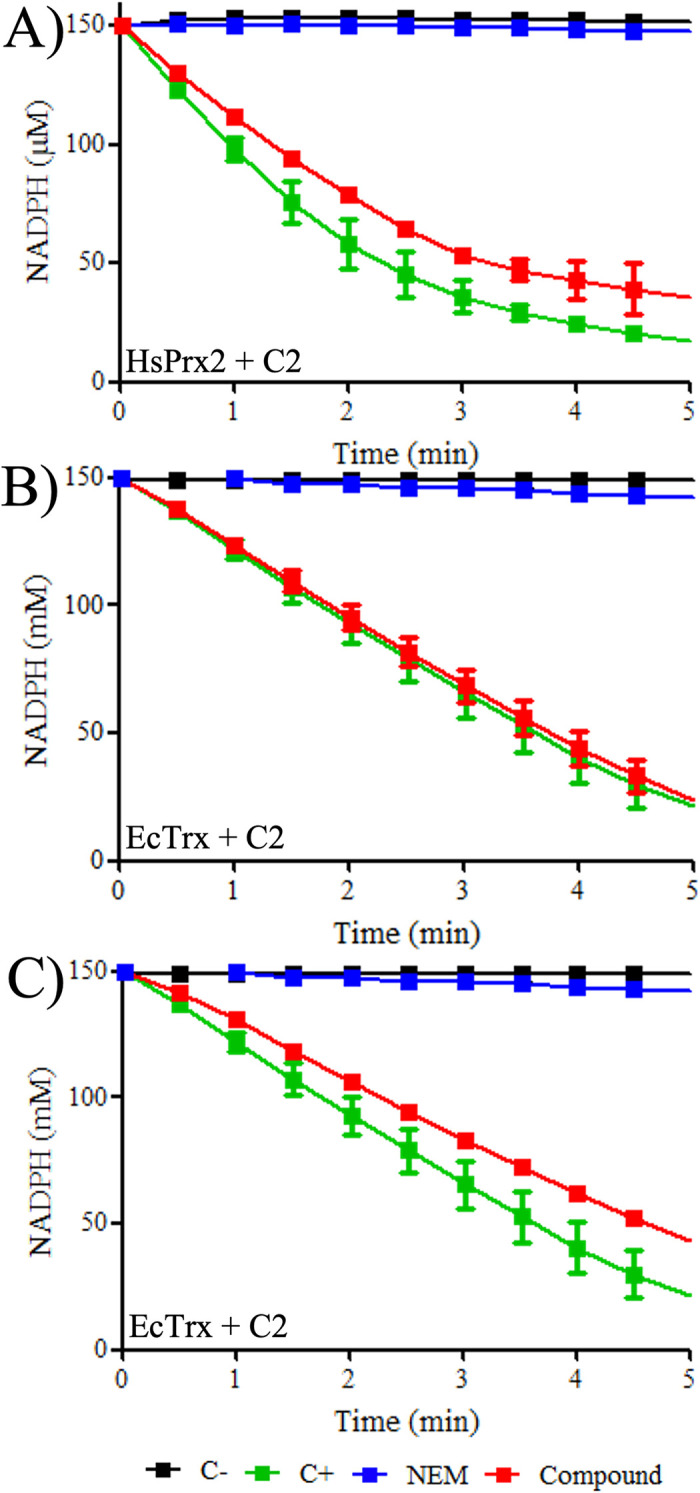
Evaluation of inhibition properties of compound C2 over HsPrx2 and the bacterial thiol proteins EcTrx and EcTrxR. The enzymes HsPrx2, EcTrx and EcTrxR were reduced with DTT and desalted, then treated with 50 molar equivalents of compound **C2** for one hour at RT (red square). The proteins were desalted again to remove excess compound and their peroxidase activity was analyzed by NADPH oxidation assay. (**A**) For Prx2, reactions were performed containing 5 μM of Prx2, 10 μM of ScTrx1, 0.3 μM of ScTrxR1, 150 μM of NADPH, 50 mM of HEPES (pH = 7.0), 100 μM of DTPA and 1 mM of sodium were used. For EcTrx (**B**) and EcTrxR (**C**) reactions consisted of 3 μM of PaAhpC, 6 μM of EcTrx, 0.9 μM of EcTrxR, 150 μM of NADPH, 50 mM of HEPES (pH = 7.4), 100 μM of DTPA and 1 mM sodium azide were used. Prior to experiments the reactions were incubated at 37°C/5 min and reactions were started by the addition of H_2_O_2_ (500 μM) and monitored spectrophotometrically at 340 nm, 37°C, for 5 minutes. The positive controls contained the proteins HsPrx2, EcTrx or EcTrxR without prior treatment with compound **C2** (green square) and the negative control without the addition of PaAhpC (EcTrx or EcTrxR) (black square). The enzymes samples previously treated with NEM were used as control of protein inhibition (blue square). The experiments were performed three times using triplicates.

We expressed and purified the HsPrx2 and used the heterologous Trx system from *S*. *cerevisiae* (ScTrx1 and ScTrxR1) (**S7E–S7G Fig**) to perform the peroxidase activity assays by NADPH oxidation assay, as described previously by Truzzi and coworkers [35]. The need for the heterologous yeast system lies in the fact that human TrxR is a seleno protein able to decompose hydroperoxides [47, 48], and the use of the human Trx system would make the results unreliable. The NADPH oxidation assay was performed using the same experimental conditions applied to the Prx bacterial isoforms, and the results revealed that compound **C2** was not able to inhibit the human isoform HsPrx efficiently, since only a very slight decrease in peroxidase activity was detected (**Fig 5A**, **Table 3**).

**Table 3:** Initial rates of peroxide decomposition after treatment with compound C2 of HsPrx2, EcTrx and EcTrxR.

Sample	No enzyme (Negative control)	Enzyme + NEM (Inhibition control)	Enzyme (Positive control)	Enzyme + C2
HsPrx2 *v*_0_ (μMs^-1^)	0.02 ± 0.01	0.02 ± 0.01	0.62 ± 0.03	0.59 ± 0.01
EcTrx *v*_0_ (μMs^-1^)	0.01 ± 0.01	0.03 ± 0.01	0.51 ±0.01	0.50 ±0.02
EcTrxR *v*_0_ (μMs^-1^)	0.01 ± 0.01	0.03 ± 0.01	0.51 ±0.01	0.49 ±0.01

Abbreviations: HsPrx2 = *H*. *sapiens* Prx2, EcTrx = *E*. *coli* Trx1 and EcTrxR = *E*. *coli* TrxR.

Concerning the thiol proteins from *E*. *coli*, compound **C2** was not able to significantly affect the activity of EcTrx or EcTrxR (**Fig 5B**). Only a very slight decay of Trx activity was detected and no inhibition was observed for EcTrxR. We then compared the residual amounts of NADPH after 300 seconds of reaction, and the results show that **C2** was not able to affect the enzymatic activity of HsPrx2, EcTrx, and EcTrxR (**S9 Fig**). These results indicate there is a selectivity of compound **C2** for PaAhpC when compared to other thiol proteins. This finding is important since some Prx inhibitors are able to inhibit other thiol enzymes, including different Prx isoforms and the Trx system enzymes [25, 26, 29, 30, 45, 46].

### Mode of PaAhpC inhibition

**Fig 6:**
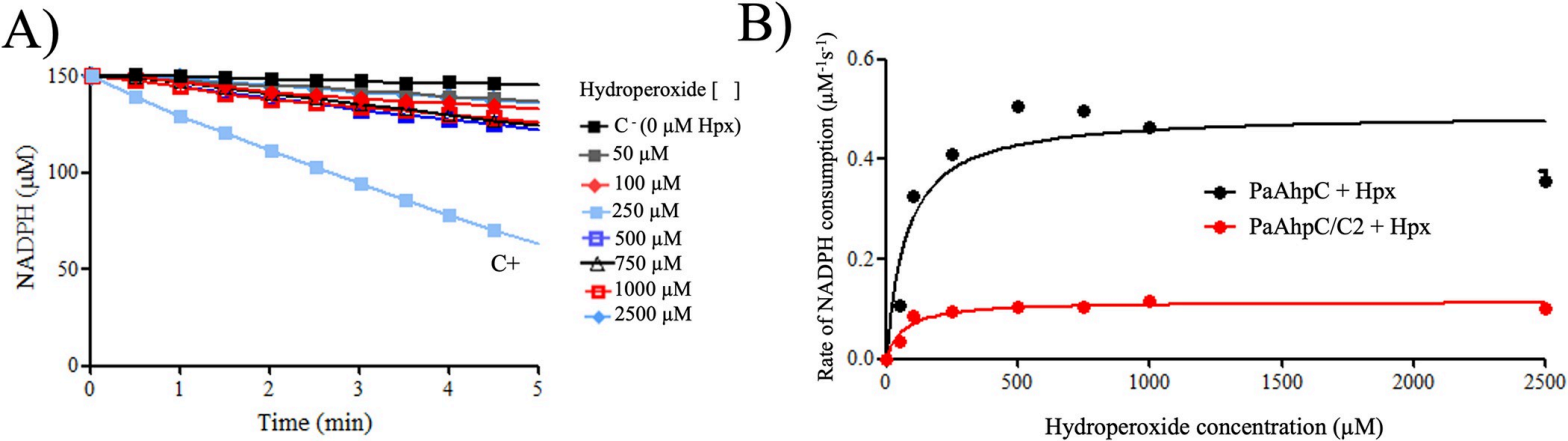
Inhibition characteristics of compound C2 over PaAhpC with growing concentrations of organic hydroperoxide. (**A**) NADPH oxidation assay of PaAhpC samples previously reduced with DTT and desalted, then treated with 20 μM of compound **C2** for one hour at RT previously treated. The reactions contained with 3 μM of PaAhpC, 6 μM of EcTrx, 0.9 μM of EcTrxR, 150 μM of NADPH, 50 mM of HEPES (pH = 7.4), 100 μM of DTPA and 1 mM sodium azide were used. Prior to experiments the reactions were incubated at 37°C/5 min and reactions were started by the addition of growing concentration of cumene hydroperoxide—CHP (50–750 μM) and monitored spectrophotometrically at 340 nm, 37°C, for 5 minutes. As negative control was used reactions without the enzyme (black square) and as positive control were used PaAhpC samples without prior treatment with compound **C2** (malibu square). The experiments were performed three times using triplicates. (**B**) Plot of the rates of NADPH oxidation by samples of pretreated with compound **C2** (PaAhpC/**C2**; red dots and line) or without inhibitor treatment (PaAhpC/**C2**; black dots and line).

To better understand the mode of inhibition, we used PaAhpC pretreated with compound **C2** (50 μM), the excess was removed and the enzyme was challenge with different concentrations of hydroperoxides (from 50 to 750 μM). Although the **C2** concentration of 50 μM is able to strongly inhibit the peroxidase activity of PaAhpC, if the mode of inhibition is a competitive behavior, the growing concentrations of peroxides may be able to shift the compound **C2** away from the active site and restore the peroxidase PaAhpC activity. Our results revealed that only a residual activity was observed (**Fig 6A**). Since the amount of inhibitor was not able to overwhelm the activity of all the enzyme population, the portion of the enzyme that had not reacted with the inhibitor would maintain its normal kinetics. The determination of kinetic parameters revealed that no significant effect was observed in the *K*_*m*_. On the other hand, parameters as *K*_*cat*_ and V_max_ were expressively impacted (**Fig 6B; Table 4**). This behavior is compatible with an irreversible inhibitor as expected by a thiol-Michael addition chemistry [29, 49–51].

**Table 4:** Enzymatic parameters determined to cumene hydroperoxide (CHP) to PaAhpC pretreated without treatment or pre-treated with compound C2.

Sample	K_*m*_ (M^-1^)	K_*cat*_ (s^-1^)	V_*max*_
PaAhpC + CHP	65.93	0.2243	0.6729
PaAhpC/**C2** + CHP	61.58	0.0765	0.1148

### Investigation of Michael addition adducts formation

Some natural products identified as inhibitors of human Prx exert their biological activity via thiol-Michael addition reaction by alkylating the catalytic cysteines [29, 30, 33]. Compounds **C1** and **C2** tested in this work possess functional groups able to perform a Michael addition, i.e., an α,β-unsaturated carbonyl group. However, while compound **C1** showed no significant inhibitory effect, compound **C2** was able to inhibit PaAhpC, suggesting that the form of inhibition may occur by other means.

To evaluate if the inhibitory mechanism of compound **C2** to PaAhpC occurs through the thiol-Michael addition, we performed non-reducing SDS-PAGE, taking advantage of the difference in gel migration of the protein in different oxidation states. When oxidized to disulfide, AhpC enzymes migrate as a dimer (~ 44 kDa) due to intermolecular disulfide bond formation. On the other hand, the reduced enzyme appears as a monomer (~ 22 kDa) [52]. Consequently, it is possible to evaluate not only the redox state of these enzymes but also if the inhibitor was able to react with cysteine residues involved in catalysis. If one of the two cysteines is covalently modified by an inhibitor, no intermolecular disulfide is expected to be formed if the enzyme is challenge with hydroperoxides, and the proteins are detected as monomers.

**Fig 7:**
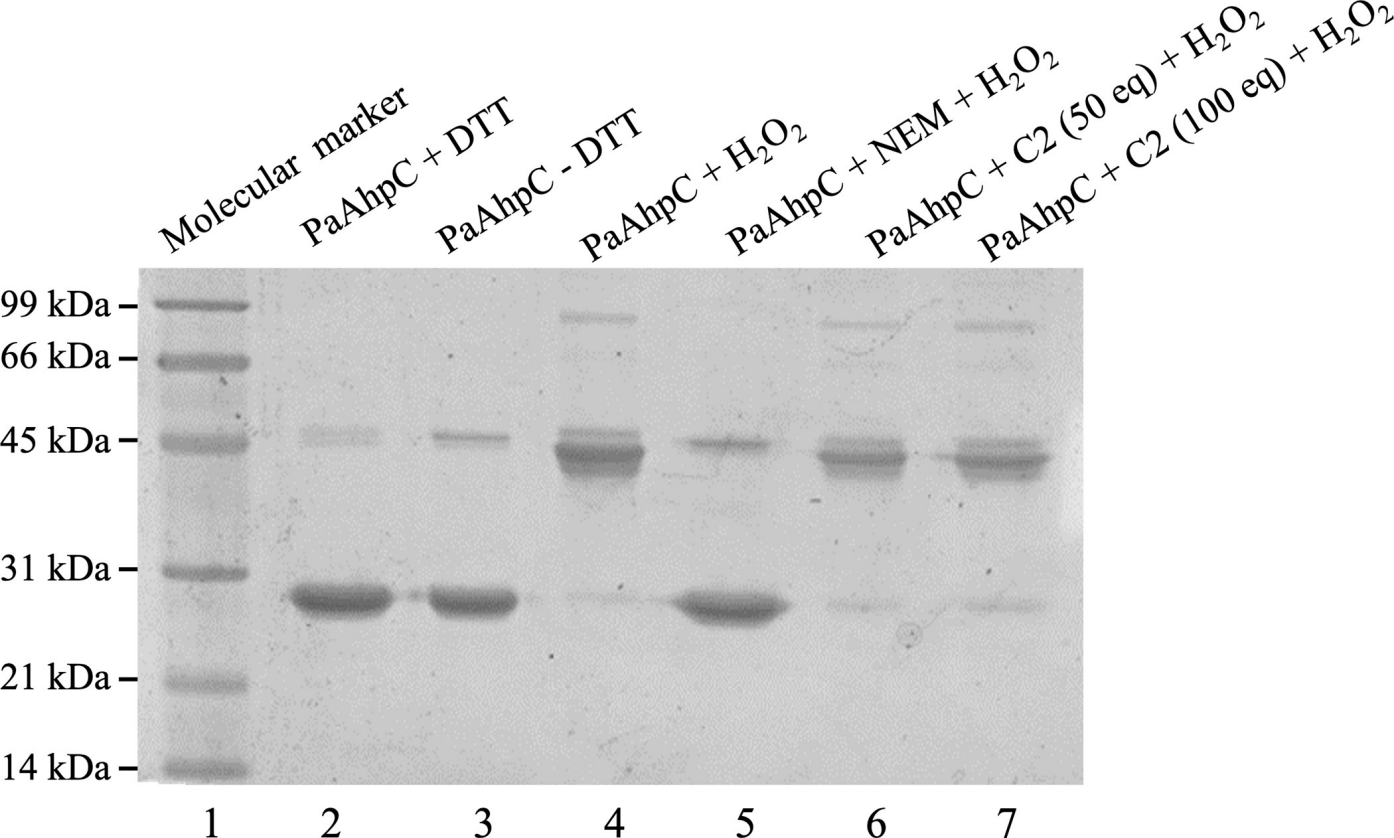
Evaluation of AhpC intermolecular disulfide formation by non-reducing SDS-PAGE. The molecular weight marker (Sigma Marker) was applied in the lane 1. Samples with 10 μM of PaAhpC were reduced with DTT 5 mM/1 h/RT (lanes 2), the excess was removed (lanes 3) and proteins were treated with 2 molar equivalents of H_2_O_2_/30 min/RT (lanes 4). As a positive control of the disulfide formation inhibition, PaAhpC samples previously reduced by DTT and desalted were treated with NEM (40 molar equivalents/1 h/RT) and then oxidized with hydrogen peroxide (30 eq.) (lanes 5). The DTT reduced and desalted samples were treated 40 and 100 molar equivalents of compound **C2** and oxidized with hydrogen peroxide (30 eq.) (lanes 6 and 7). The experiments were performed three times presenting similar results.

Prior to peroxide treatment, PaAhpC samples were reduced by DTT (**Fig 7, lane 2**), the excess was removed (**Fig 7, lane 3**), and the PaAhpC proteins were oxidized with hydrogen peroxide (**Fig 7, lane 4**). As expected, PaAhpC reduced by DTT were detected as monomers, while the oxidized samples were detected as dimers. As internal control of the inhibition of the disulfide formation, PaAhpC sample was previously treated with NEM and then oxidized by hydrogen peroxide.

The NEM treatment prevented disulfide formation and the proteins migrated as monomers (**Fig 7, lane 5**). Regarding samples pre-treated with compound **C2** (**Fig 7, lanes 6 and 7**), the PaAhpC samples were also detected as dimers, even at very high concentration (e.g. 100 molar equivalents), revealing the formation of the intermolecular disulfide and indicating the mode of inhibition does not occur by the Michael addition mechanism over the protein thiols. This suggests that compound **C2** is able to inhibit PaAhpC in a manner distinct from those already described for other Prx inhibitors from eukaryotes [29, 30, 33] and bacteria [25, 26]. Although our data suggests that the PaAhpC cysteine is not alkylated by compound **C2**, another reactive groups as amine (-NH_2_) and alcohol (-OH) can perform the Michael addition chemistry (e.g. aza- and oxa-Michael addition, respectively) [53, 54]. Both groups are present in the side chains of the PaAhpC catalytic triad residues Arg and Thr and are involved in substrate targeting and stabilization (**Fig 1**) and the modification of either group by **C2** may have a strong impact in enzyme activity.

### Molecular docking approaches

**Fig 8:**
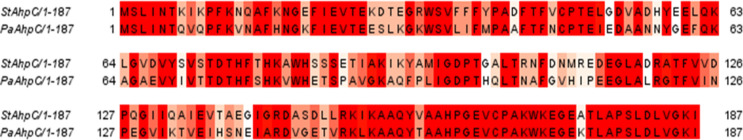
Alignment of the amino acid sequences of AhpCs from *S*. *typhimurium* and *P*. *aeruginosa*. The alignment of AA sequences was performed using Clustal Ω and the figure was generated with the Jalview. The abbreviations used and access codes for Uniprot (www.uniprot.org) are: AhpC of *S*. *typhimurium* = StAhpC (Uniprot: P0A251) and AhpC of *P*. *aeruginosa* = PaAhpC (Uniprot: Q02UU0).

To assess molecular interactions between compound **C2** and PaAhpC, molecular docking simulations were performed using the structure of AhpC from *S*. *typhimurium*, since AhpC from *P*. *aureginosa* (PaAhpC) has no determined crystallographic structure. The *P*. *aeruginosa* and *S*. *typhimurium* isoforms are highly related, presenting 75% of similarity and 60% identity (**Fig 8**).

**Fig 9:**
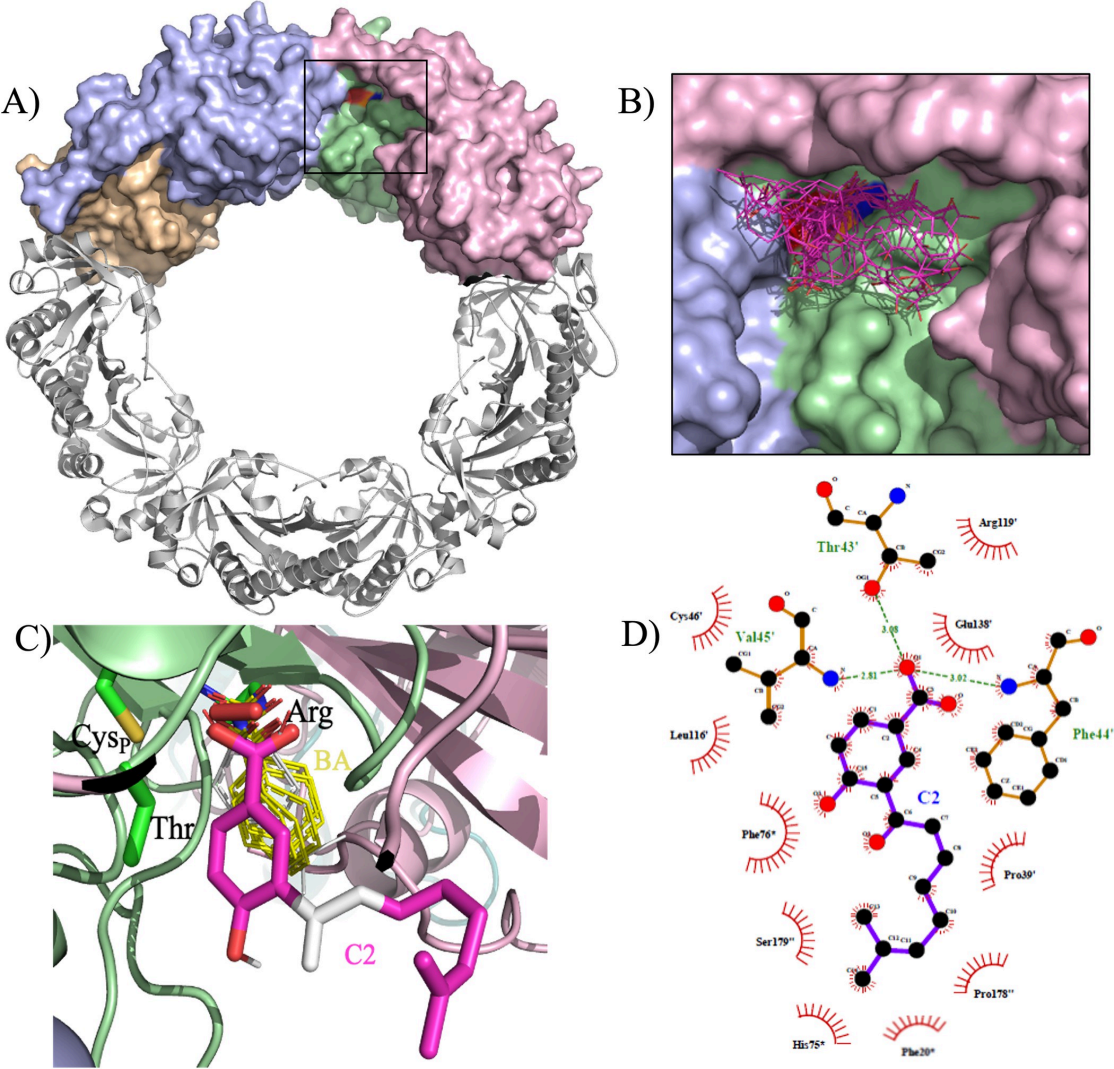
Decameric structure of AhpC and docking results and best pose selection based in peroxiredoxin ligands found in pdb database. (**A**) *S*. *typhimurium* AhpC (PDB = 4MA9) in reduced decameric state. The enzyme is composed by five obligate homodimers and the two upper homodimers are represented by molecular surface and colored in purple/beige and green/pink. The other dimers of the decamer are represented in cartoon and colored in light gray. The black box denotes the active site microenvironment located at the dimers interface. The catalytic triad residues Thr, Cys_P_ and Arg are colored in red, orange and blue, respectively. (**B**) Molecular docking results for compound **C2**. The molecules cluster of the compound **C2** are represented by lines and colored in pink. (**C**) Superposition of the compound **C2** best hit to StAhpC with ligands found in Prx structures deposited in the pdb database: H_2_O_2_ (PDB code = 3A2V; color = red), benzoate (2V32; 1HD2; 1OC3; 2V41; 1H4O; yellow) and tert-butylbenzene-diol (4K7O; white). The **C2** is colored in pink with the atoms of α,β-unsaturated carbonyl system highlighted in white. The other molecules are represented in lines with different colors. The oxygens are in red. The catalytic triad (Thr, Cys_P_ and Arg) is represented by sticks. **(D)** Diagram of interacting residues of AhpC with compound **C2** docked in the StAhpC active site. The dashed green lines represent hydrogen bonds and hydrophobic contacts are represented by red arcs with radiating spikes. The binding analysis was performed using the LigPLot^+^.

Docking approaches were performed using the decameric structure once the decamer is in the active form, whose geometry is optimal for the productive binding of the peroxide substrates. The AhpC active site environment is composed of residues of three protomers: the obligate dimer and the monomer of the neighboring dimer (**Fig 9A**).

The solutions were distributed mostly in the microenvironment of the active site in close proximity to the residues involved in catalysis (**Fig 9B**). The best pose was selected based on the free energy of Gibbs (ΔG = -7.2 kcal/mol) and superposition with ligands found in the active site of Prx crystallographic structures, such as the oxidant substrate (hydrogen peroxide in *Aeropyrum pernix* Prx) [55] and compounds with structural similarities to **C2** compound (benzoic acid found in the *Arenicola marina* Prx6 and *Homo sapiens* Prx5 and tert-butylbenzene-diol present *H*. *sapiens* Prx5) [56–58] (**Fig 9C**).

Stabilization of compound **C2** mostly occurred by hydrophobic interactions and van der Waals forces interactions from the main or side chains (MC or SC) of the interacting from obligate dimer: P^39’^ (SC), T^43’^(SC), F^44’^(MC), V^45’^ (MC) (N^45’^, in PaAhpC), C_P_^46’^ (MC), L^116’^(SC), R^119’^(SC), E^138’^(SC) (N^138^, in PaAhpC), P^178”^ (SC/MC), and S^179”^ (MC); and from the monomer of the adjacent dimer: F^20^*(SC), H^75^* (SC), and F^76^*(SC) (the prime (‘) and quotation (”) symbols denotes residues form the obligate dimer, and the asterisk (*) denotes the adjacent protomer dimer) (**Fig 9D**).

The functional importance of some residues identified in the docking procedures has been determined. It has already been shown that the Phe/Tyr residue at this position in peroxiredoxins is involved in the decamer stabilization of the enzyme, through an unconventional hydrogen bond with the catalytic triad Thr [22, 23, 59, 60]. Additionally, Glu^138^ would be related to conformational changes during the catalytic cycle [61]. Except for the Val^45^ and Glu^138^ of StAhpC that are substituted by Asn in PaAhpC, a residue with similar physicochemical properties, all the remaining interacting residues are strictly conserved among the two peroxiredoxins, which corroborates the feasibility of the docking analysis.

An important aspect about the mode of inhibition, which was irreversible, may have been related to the adoption of Michael with other groups such as the O and N atoms from the side chain of the catalytic triad Thr of Arg residues. The α,β-unsaturated carbonylic system is in close proximity to both reactive group residues of these amino acids, which are also more exposed than Cys_P_ and involved in interactions to guide the substrates to catalytic cysteines (**Fig 1**). Nonetheless, these assumptions lack experimental support and the determination of PaAhpC structures with compound **C2** is needed to reach any meaningful conclusions.

## Discussion

We tested two biosynthetic related natural products isolated from *P*. *crassinervium* as inhibitor of the AhpC from bacteria: 4-hydroxy-2-(3’,7’-dimethyl-1’-oxo-2’*E*,6’-octadienyl) benzoic acid (**C1**), and 4-hydroxy-2-(3’,7’-dimethyl-1’-oxo-2’*E*,6’-octadienyl) benzoic acid (**C2**). Both compounds possess three common structural aspects: 1) benzene rings, 2) an α,β unsaturated carbonylic system, and 3) a flexible tail (oxo-geranyl unity) that resembles long-chain fatty acids. The sole difference is a hydroxyl group at C-4 position in compound **C1** which is substituted by a carboxylic acid in compound **C2** (**Fig 2**). Therefore, it is reasonable to assume that the carboxyl group is involved in the inhibitory properties of compound **C2**. In fact, benzoic acids and derivatives have been found in some crystallized in different peroxiredoxin structures, suggesting that the carboxylic acid in the aromatic ring is a factor that may favor the stabilization of molecules in the active site [56, 57, 62].

The hydrophobic nature, including the presence of one aromatic ring, places compounds **C1** and **C2** with other chemically related molecules identified for this group of proteins [29, 30, 32, 33, 63, 64]. Although the oxo-geranyl unity has not been observed in previously described inhibitors, it resembles substrates such as long-chain fatty acids hydroperoxides (oleic and linoleic acid peroxides, for example) which are believed to be biological substrates of some thiol peroxidases including Prx [65, 66]. The docking approaches presented here reveal that the tail may be stabilized in the active site pocket by several hydrophobic interactions with non-polar residues. This feature appears to be important and can be exploited in the search for natural inhibitors for Prx. Unlike other inhibitors that also present an α,β-unsaturated carbonyl system, neither compound **C1** and **C2** perform a thiol-Michael addition reaction over the reactive cysteines, revealing a mode of inhibition distinct from those previously characterized with other natural compounds to the human isoforms [29, 30]. This is an important aspect since most of the Prx inhibitors described to date perform an inhibition by means of chemical reactions involving a covalent bond between ligand and the thiol of the cysteines but with low specificity, affecting other thiol proteins and being very toxic to the cells [25, 45, 46]. Conversely, the compound **C2** shows high specificity over PaAhpC when compared to SeAhpC, HsPrx, or even other thiol proteins as the Trx system which are strongly inhibited by other Prx inhibitors [26, 45, 46]. The specificity over PaAhpC is a very positive finding, since this enzyme is a *P*. *aeruginosa* virulence factor, and this pathogenic bacterium is related with nosocomial infections and high mortality rates [67, 68]. In summary, our results demonstrate the inhibitory activity of a new class of natural products that possesses distinct characteristics from other compounds identified as peroxiredoxin inhibitors. This contribution on molecules able to exert inhibitory activity on Prx of bacteria can allow the selection of natural products or even lead to the synthesis of inhibitors, aiming to increase the specificity and inhibitory activity over the Prx in order to combat infectious and genetic diseases.

## Supporting information

S1 Fig ^1^H NMR spectrum of compound C1 (δ, CDCl3, 500 MHz). (TIFF)

S2 Fig ^13^C NMR spectrum of compound C1 (δ, CDCl3, 125 MHz). (TIFF)

S3 Fig ESI-HRMS of compound C1 (negative mode). (TIFF)

S4 Fig ^1^H NMR spectrum of compound C2 (δ, CDCl3, 500 MHz). (TIFF)

S5 Fig ^13^C NMR spectrum of compound C2 (δ, CDCl3, 125 MHz). (TIFF)

S6 Fig ESI-HRMS of compound C2 (negative mode). (TIFF)

S7 Fig Purification by IMAC of recombinant enzymes PaAhpC (A), SeAhpC (B), EcTrx (C), EcTrxR (D), HsPrx2 (E), ScTrx1 (F) and ScTrxR (G) expressed in *E*. *coli* BL21 (DE3). Reducing SDS-PAGE (200mM—mercaptoethanol, 12% polyacrylamide) representative of purification results of recombinant His-tag-containing proteins purified by IMAC (A, B, C, D, E, F, G) (using HiTrap TALON crude column (Cytiva Life Sciences) or by the boiling method (F). The black arrow denotes the position of the recombinant protein in the gel.
(TIFF)

S8 Fig Residual NADPH after 300 seconds of treated and untreated PaAhpC and SeAhpC with compounds C1 and C2 or alkylating agent (NEM). The amount of NADPH consumed after 300 s of reaction were obtained in the assays presented in Fig 3. The graph shows final amount of NADPH at the end of the assay. The bars are colored as follows: black square = negative control (without enzyme); green square = PaAhpC (positive control), blue square = NEM treated PaAhpC (peroxidase inhibition control), red square = PaAhpC + **C1**, PaAhpC + **C2**, SeAhpC + **C1** or SeAhpC + **C2**. Assays were performed in triplicate and repeated at least three times.
(TIFF)

S9 Fig Residual NADPH after 300 seconds of untreated and treated HsPrx2, EcTrx and EcTrxR with compound C2 or alkylating agent (NEM). The amount of NADPH consumed after 300 s of reaction were obtained in the assays presented in Fig 5. The graph shows final amount of NADPH at the end of the assay. The bars are colored as follows: black square = negative control (without enzyme); green square = HsPrx2, EcTrx or EcTrxR (positive control), blue square = NEM treated enzyme (peroxidase inhibition control), red square = HsPrx2, EcTrx or EcTrxR + **C2**. Assays were performed in triplicate and repeated at least three times.
(TIFF)
