# Supplementary figures and images for: Exploring functional and structural features of chemically related natural prenylated hydroquinone and benzoic acid from Piper crassinervium (Piperaceae) on bacterial peroxiredoxin inhibition
Source: PLoS One. 2023 Feb 24;18(2):e0281322. doi: 10.1371/journal.pone.0281322 (PMC9956870; doi:10.1371/journal.pone.0281322)

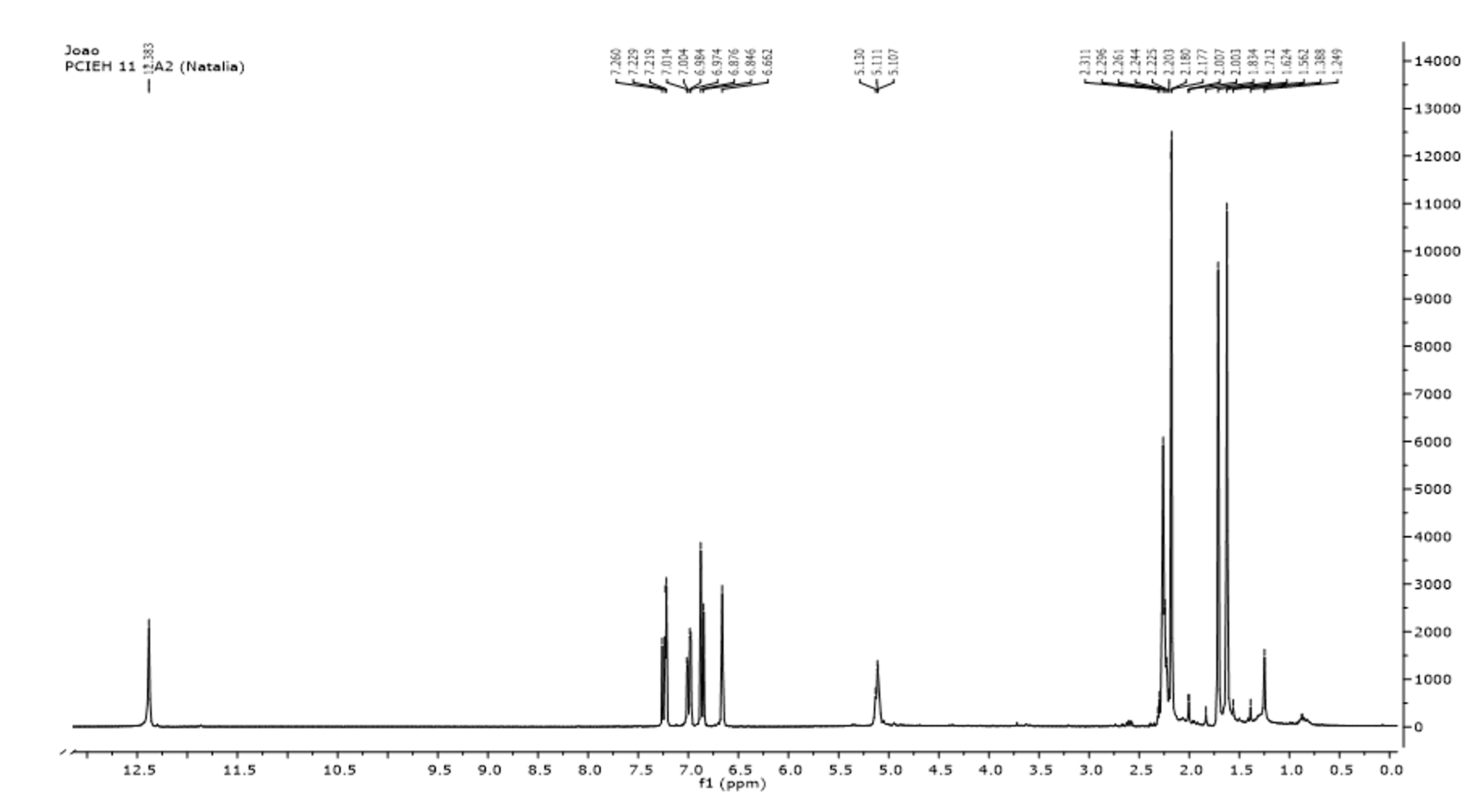

Supplement: S1 Fig — (TIFF) [file pone.0281322.s001.tiff]

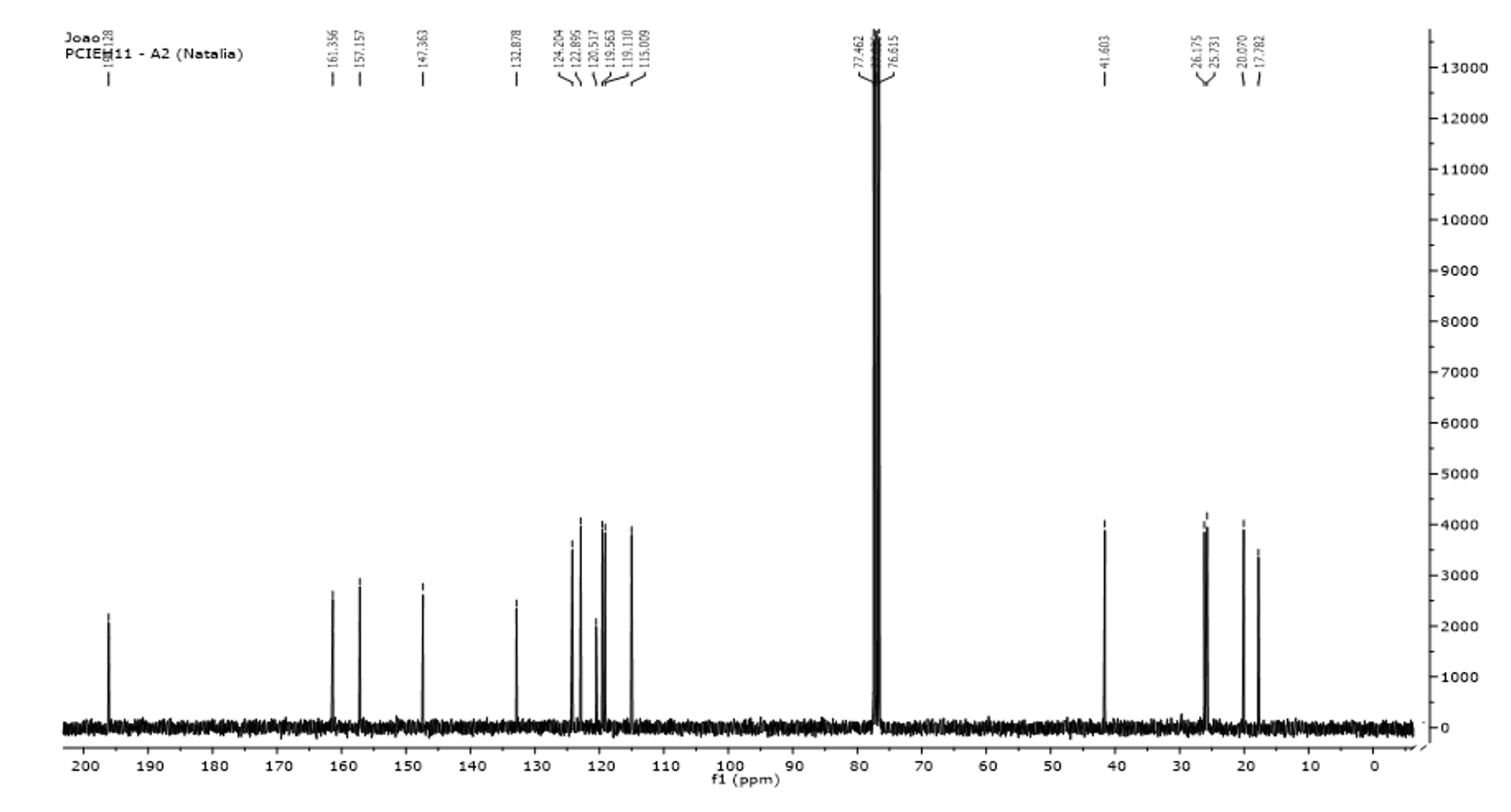

Supplement: S2 Fig — (TIFF) [file pone.0281322.s002.tiff]

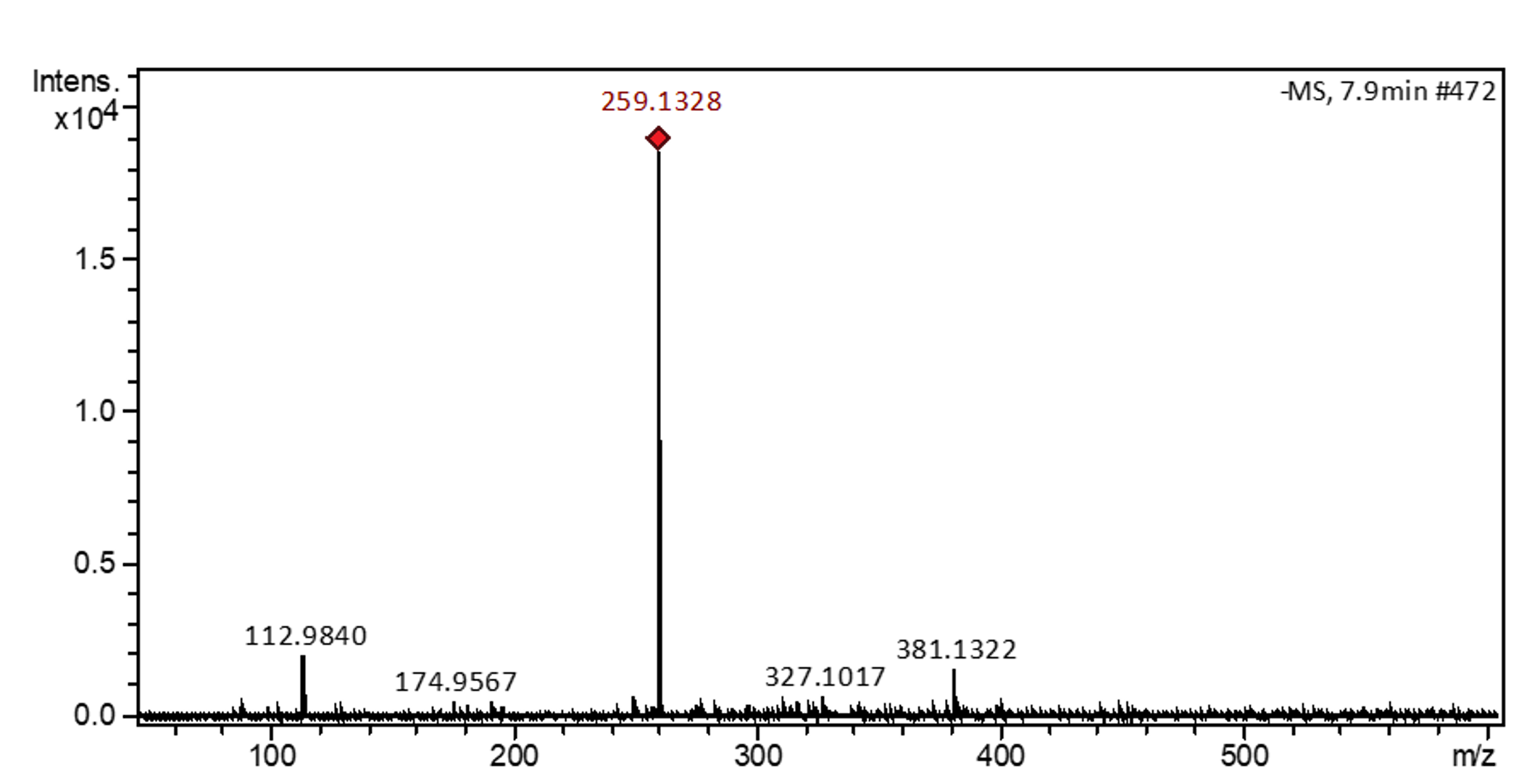

Supplement: S3 Fig — (TIFF) [file pone.0281322.s003.tiff]

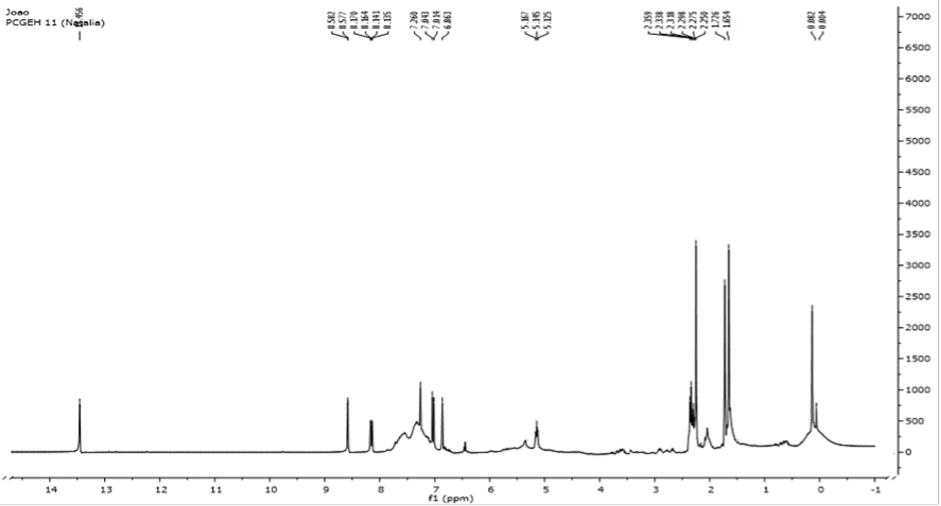

Supplement: S4 Fig — (TIFF) [file pone.0281322.s004.tiff]

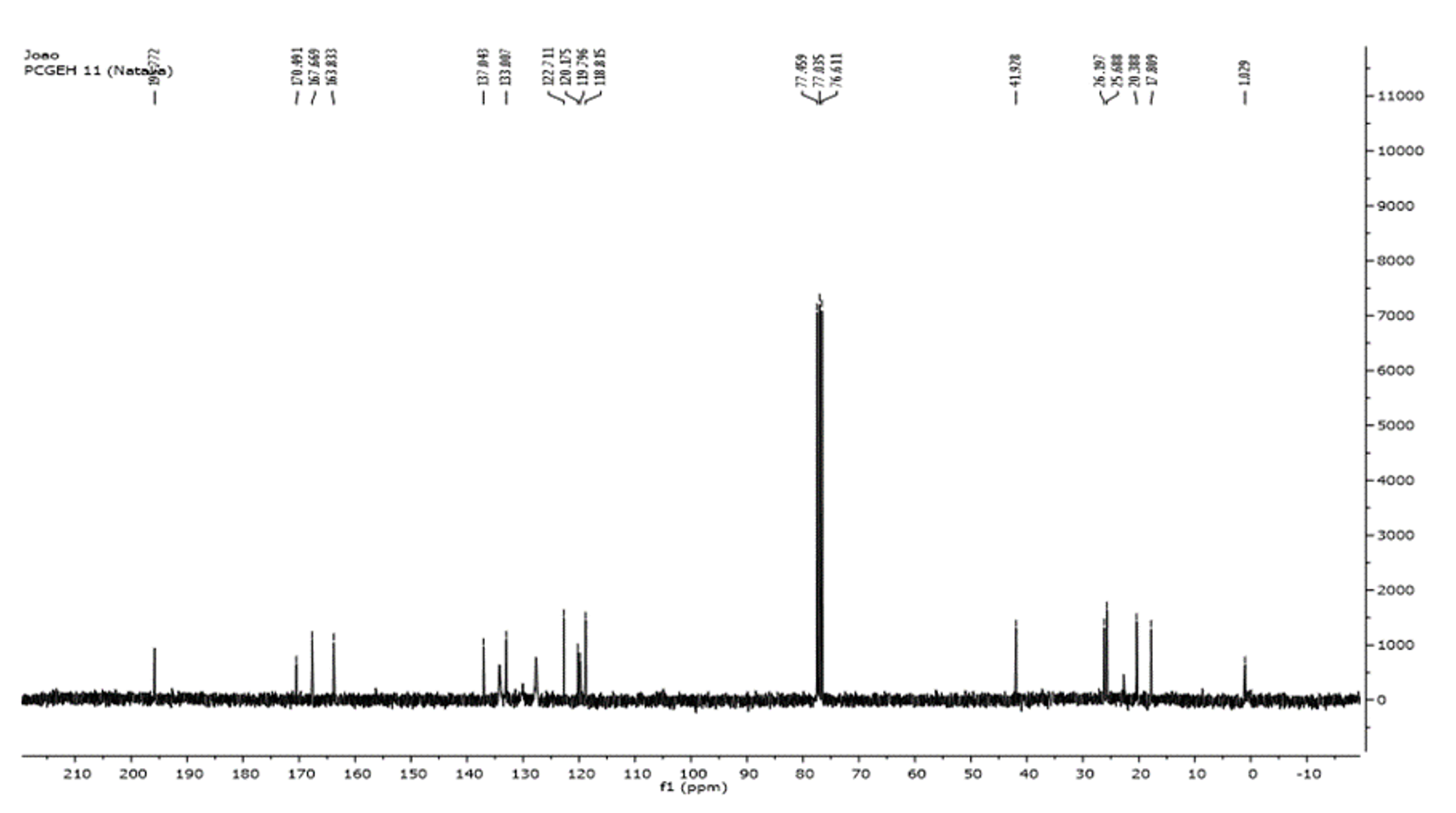

Supplement: S5 Fig — (TIFF) [file pone.0281322.s005.tiff]

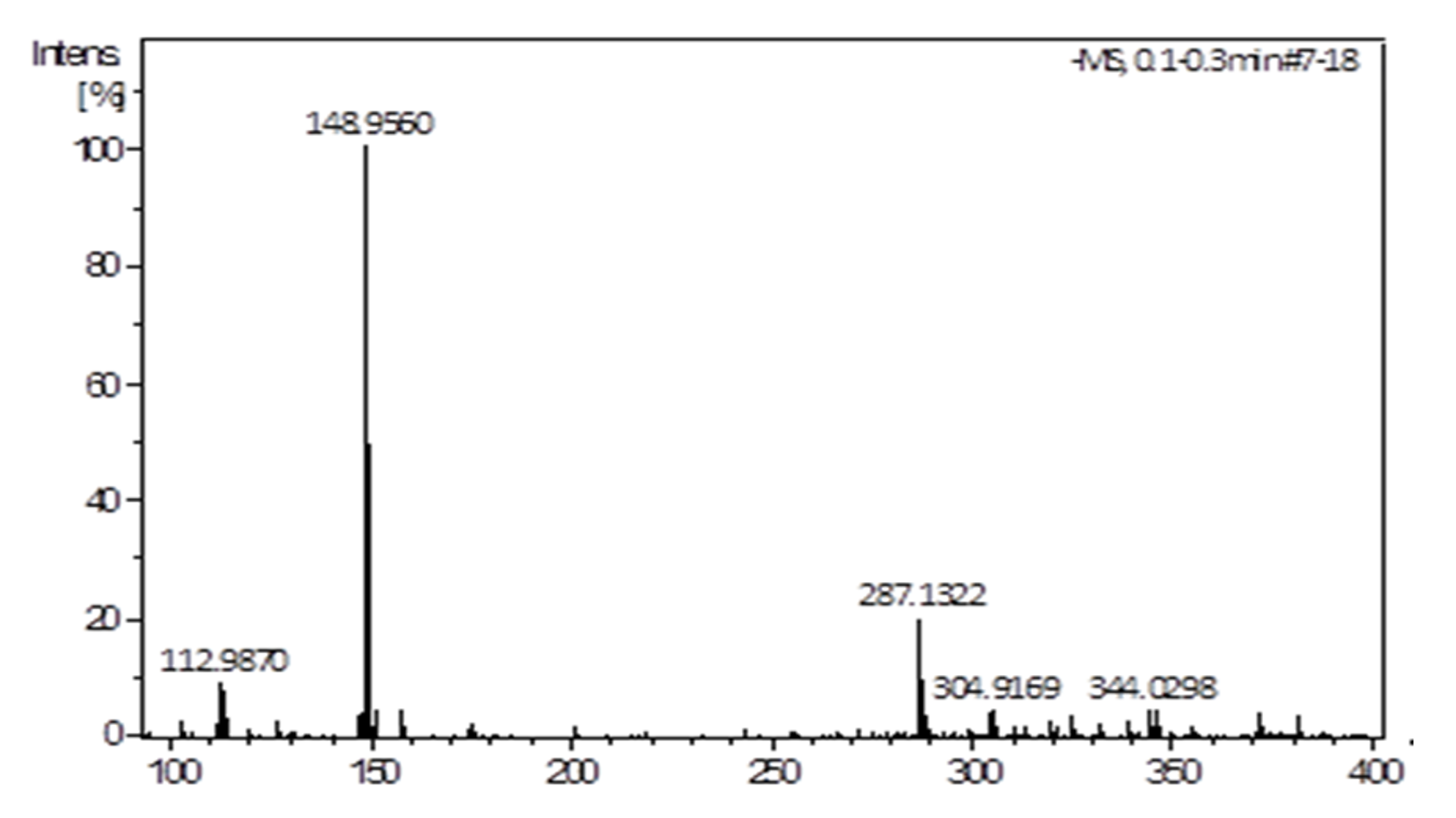

Supplement: S6 Fig — (TIFF) [file pone.0281322.s006.tiff]

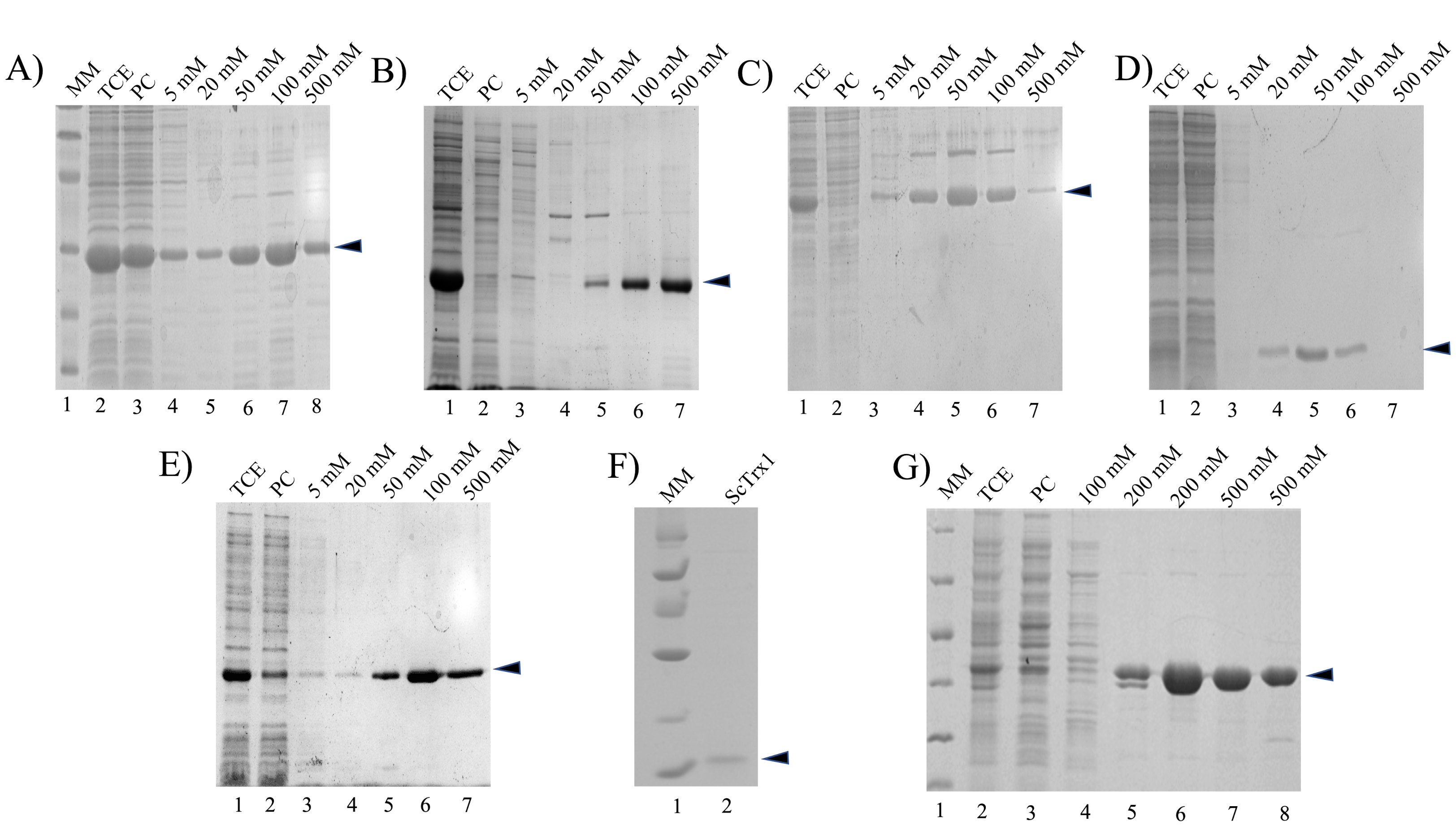

Supplement: S7 Fig — Purification by IMAC of recombinant enzymes PaAhpC (A), SeAhpC (B), EcTrx (C), EcTrxR (D), HsPrx2 (E), ScTrx1 (F) and ScTrxR (G) expressed in E. coli BL21 (DE3). Reducing SDS-PAGE (200mM—mercaptoethanol, 12% polyacrylamide) representative of purification results of recombinant His-tag-containing proteins purified by IMAC (A, B, C, D, E, F, G) (using HiTrap TALON crude column (Cytiva Life Sciences) or by the boiling method (F). The black arrow denotes the position of the recombinant protein in the gel. (TIFF) [file pone.0281322.s007.tiff]

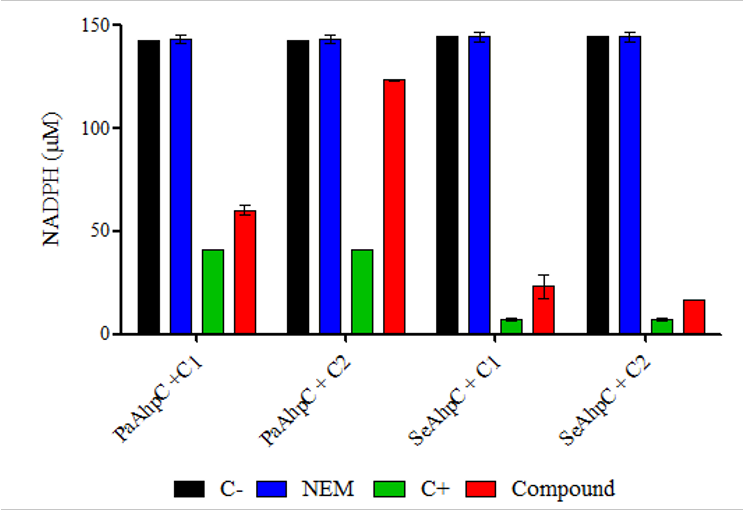

Supplement: S8 Fig — The amount of NADPH consumed after 300 s of reaction were obtained in the assays presented in Fig 3. The graph shows final amount of NADPH at the end of the assay. The bars are colored as follows: black square = negative control (without enzyme); green square = PaAhpC (positive control), blue square = NEM treated PaAhpC (peroxidase inhibition control), red square = PaAhpC + C1, PaAhpC + C2, SeAhpC + C1 or SeAhpC + C2. Assays were performed in triplicate and repeated at least three times. (TIFF) [file pone.0281322.s008.tiff]

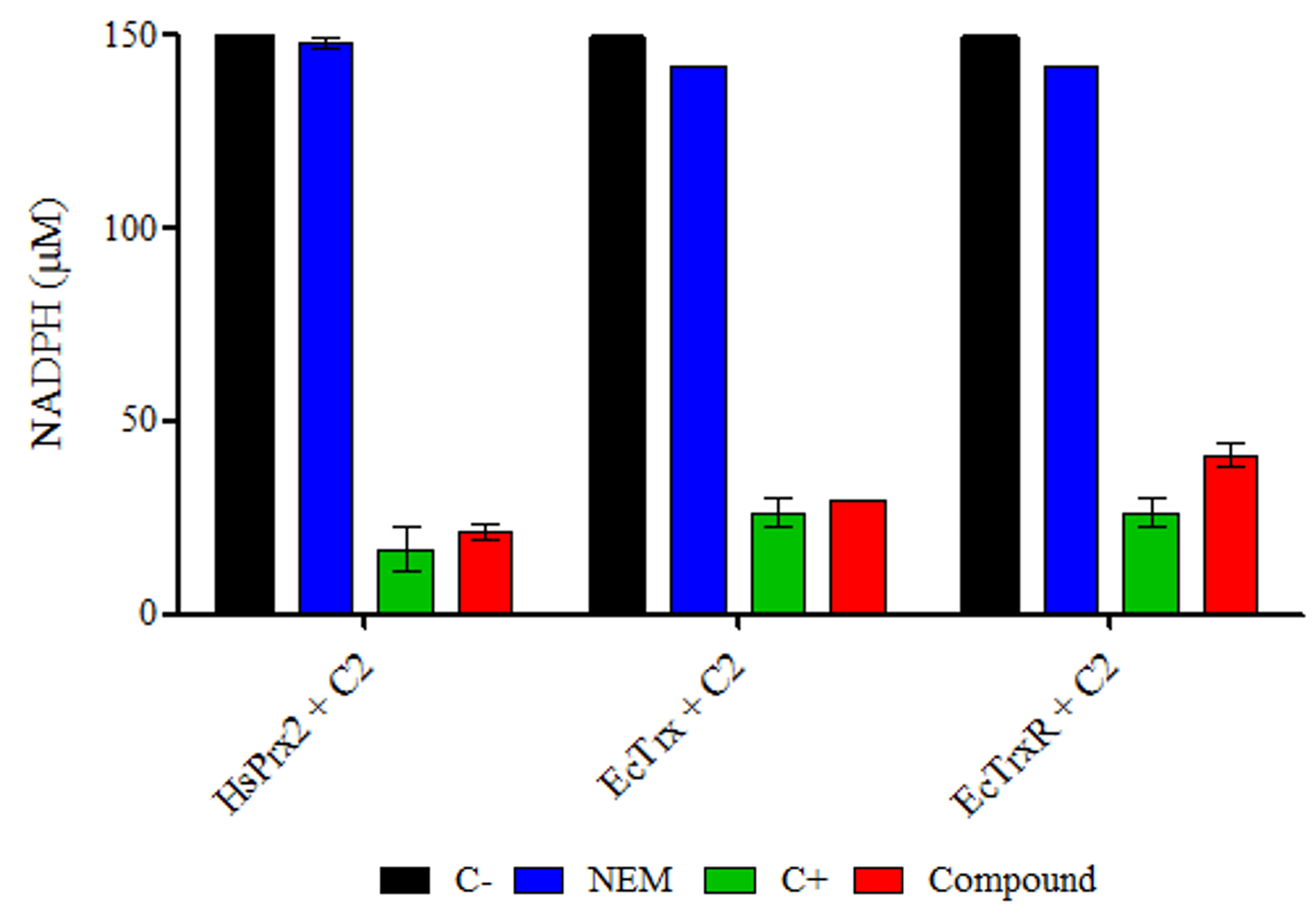

Supplement: S9 Fig — The amount of NADPH consumed after 300 s of reaction were obtained in the assays presented in Fig 5. The graph shows final amount of NADPH at the end of the assay. The bars are colored as follows: black square = negative control (without enzyme); green square = HsPrx2, EcTrx or EcTrxR (positive control), blue square = NEM treated enzyme (peroxidase inhibition control), red square = HsPrx2, EcTrx or EcTrxR + C2. Assays were performed in triplicate and repeated at least three times. (TIFF) [file pone.0281322.s009.tiff]
